# When is “convenient time” for residents?: a trial of Resident Study Log as asynchronous learning tool for residents

**DOI:** 10.1080/10872981.2025.2461579

**Published:** 2025-02-02

**Authors:** Tadayuki Hashimoto, Shoko Ariyoshi, Taira Ariyoshi, Ryosuke Horitani, Mohammad Adrian Hasdianda, Kanapa Kornsawad, Shunsuke Kosugi, Makoto Kikukawa, Tomio Suzuki

**Affiliations:** a Department of Emergency Medicine, Brigham and Women’s Hospital, Boston, MA, USA; b Department of General Medicine, Osaka Medical and Pharmaceutical University, Takatsuki, Japan; c Department of General Internal Medicine, Hashimoto Municipal Hospital, Hashimoto, Japan; d Division of Hospital Medicine, Department of Medicine, University of Texas Health Science Center at San Antonio, San Antonio, TX, USA; e Department of Medical Education, Faculty of Medical Sciences, Kyushu University, Fukuoka, Japan

**Keywords:** Asynchronous online learning, postgraduate medical education, self-reflection, digital learning tools, well-being, sleep hygiene, work-life balance in residency

## Abstract

**Introduction:**

The integration of online learning in health professions education has grown rapidly, offering flexibility to learners worldwide. Asynchronous online learning allows residents to engage with educational content at a time convenient for them, accommodating their demanding schedules. This study aims to reveal how residents approach asynchronous online learning during their residency training.

**Methods:**

The Resident Study Log, an asynchronous learning tool, was introduced in a teaching hospital in Japan. This tool facilitated residents to share daily reflections on their learning experiences, allowing supervising physicians outside of their regular clinical teams to provide feedback. Posts shared between April 2020 and July 2021 were reviewed. The outcome measured included the frequency and timing of posts as well as residents’ satisfaction and perceived burden, which were assessed through anonymous surveys.

**Results:**

A total of 31 residents participated during the study period, posting 599 entries on the Resident Study Log, averaging one post per resident every two days. Participants reported an average satisfaction score of 5.3 out of 6.0 (6-point Likert scale, where 1 indicated ‘not satisfied at all’ and 6 indicated ‘very satisfied’), while the perceived burden averaged 4.0 out of 6.0 (6-point Likert scale, where 1 indicated ‘not burdensome at all’ and 6 indicated ‘very burdensome’). Notably, posting activity peaked at around 23:00, with 17.4% of posts created between midnight and 5:00.

**Conclusion:**

The Resident Study Log was a simple, low-cost tool, with high satisfaction levels among residents. However, the late-night posting pattern raises concerns about resident well-being and sleep hygiene. As asynchronous learning is increasingly more prevalent, it is crucial to ensure it does not inadvertently impose hidden burdens on learners’ overall well-being.

## Introduction

In recent times, the intensity of clinical training for medical trainees, such as residents, has raised concerns regarding their well-being [1]. In response, various global initiatives have implemented work-hour restrictions to safeguard their health [1–4]. These restrictions, while necessary, introduce challenges to the traditional model of medical education, which must now adopt more flexible and innovative approaches to ensure the effectiveness of training [1].

In parallel, the integration of online learning within health professions education has been recognized as a valuable tool for advancing medical knowledge and skills [5]. Specifically, asynchronous online learning offers the unique advantage of allowing learners to engage with the content at their convenience [6,7]. This flexibility is especially advantageous for health professionals, including medical residents, who must balance demanding clinical schedules with the need for continuous learning [5].

The alignment of postgraduate medical education with asynchronous online learning presents a promising avenue for addressing the challenges posed by work-hour restrictions [5]. Therefore, exploring how medical residents engage with this learning format is critical. This study aims to investigate residents’ approaches to asynchronous online learning by examining the frequency and timing of their posts, as well as their perceptions of the experience, through anonymous surveys.

## Materials and methods

### Setting and participants

Hashimoto Municipal Hospital, a 300-bed acute care teaching facility in Hashimoto, Japan, has a Department of General Internal Medicine (DGIM) staffed by three supervising physicians and approximately three residents on two-month rotations. Each team comprises one supervising physician and one resident.

### Development of an asynchronized learning method

To address the lack of interaction with other teams and to foster self-reflection and peer learning, we developed the Resident Study Log, an asynchronous learning tool to help residents track their learning, promote self-reflection, and receive feedback from other clinicians. The Resident Study Log was implemented via Slack, a widely used digital workspace that supports asynchronous collaboration [8]. Several initiatives have emerged that leverage Slack in the context of medical education [9–11]. Residents were instructed to post daily reflections on Slack, with supervising physicians providing feedback. Working hours were from 8:30 to 17:15, with no on-call or night shifts required. Orientation introduced residents to the system, and automatic Slack reminders at 17:00 encouraged posting. Regarding monitoring, all supervising physicians participated in reviewing posts. However, as the primary developer of the system, the first author was responsible for ensuring oversight by checking the posts before the start of the next day’s work hours.

This method was independently developed by the first author, inspired by the personal practice of reflective learning over the years. By leveraging Information and Communication Technology (ICT), the aim was to promote peer learning while maintaining flexibility. Taking advantage of the unique characteristics of General Internal Medicine, the residents were encouraged to post about any topic, including not only medical knowledge but also social and psychiatric aspects. Participants were allowed to write entries as brief as a single line.

Although online education platforms are typically costly [12,13], this implementation incurred no costs, utilizing an existing Slack account. The Slack account was created using the DGIM’s official email account, and a free account was utilized for this implementation. Participants were instructed to create their own individual free Slack accounts on the first day of their rotations, enabling the system to operate entirely free of charge.

### Outcome measures

We conducted a cross-sectional study and reviewed the posts collected over a 16-month study period from 1 April 2020, to 31 July 2021. We calculated the frequency and timing of the residents’ posts. Resident satisfaction (assessed using a 6-point Likert scale, where 1 indicated ‘not satisfied at all’ and 6 indicated ‘very satisfied’) was measured with the question, ‘Were you satisfied with using Slack for the Resident Study Log during your training?’ Similarly, perceived burden levels (assessed using a 6-point Likert scale, where 1 indicated ‘not burdensome at all’ and 6 indicated ‘very burdensome’) were measured with the question, ‘Did using Slack for the Resident Study Log feel burdensome during your training?’ These questions were part of an anonymous survey administered at the end of each rotation as a component of the overall rotation evaluation form. Data from the survey responses were compiled into Microsoft Excel. For each question, the mean Likert score and standard deviation were calculated using Excel’s built-in statistical functions to summarize central tendency and variability. This study was approved by the Hashimoto Municipal Hospital Research Ethics Committee (#R3.7–6).

## Results

**Figure 1.**
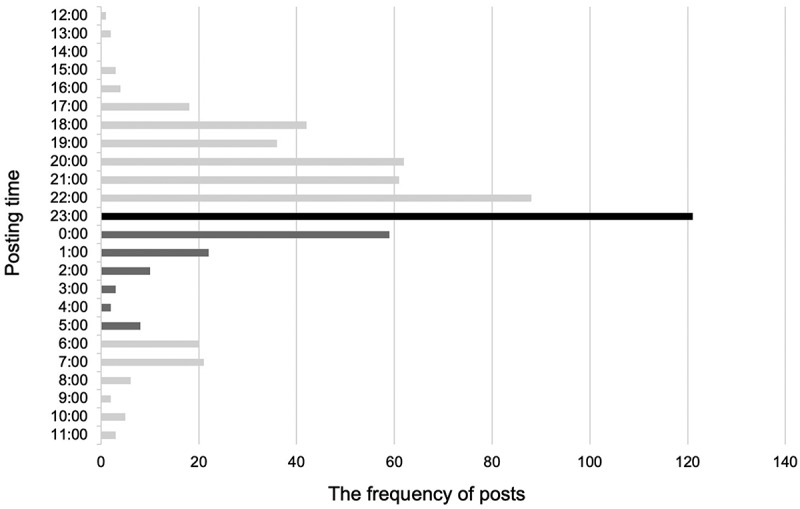
Daily posting patterns by hour.

A total of 31 residents (11 in their 1^st^ postgraduate year and 20 in their second) rotated during the study period. The residents shared a total of 599 posts on the Resident Study Log, with an average of one post every two days per resident. Although residents were instructed that posts could be as brief as a single line, all posts spanned multiple lines. The mean score of their satisfaction was 5.3 out of 6.0 (±0.61 SD) and the mean score of their burden was 4.0 out of 6.0 (±0.97 SD). The time of posting peaked between 23:00 and midnight (121 times), and a total of 104 (17.4%) were posted between midnight and 5:00 (Figure 1). Each post received feedback from at least one supervising physician. If the content required corrections or additional explanations, comments were added; otherwise, a ‘like’ button was used.

## Discussion

The Resident Study Log demonstrated itself to be a simple and sustainable teaching tool with relatively high resident satisfaction. Despite minimal intervention, residents posted approximately once every two days, more frequently than initially expected. In this regard, it can be considered that the tool successfully fostered self-reflection.

However, the most notable finding in this study is the timing of the residents’ posts with a significant number of posts shared late at night. Previous research highlights that asynchronous learning allows learners to study at their own pace and at times most convenient for them [5]. There are several potential drawbacks, such as distractions, delayed learning, a lack of immediate, just-in-time feedback, learner disengagement due to reduced social interaction, high development costs, technical problems, and issues arising from poor instructional design [14,15]. Curiously, previous studies do not suggest that asynchronous learning inherently burdens learners in terms of time commitment.

The well-being of physicians, including residents, is a critical issue and it is significantly influenced by their amount and quality of sleep [16,17]. Poor sleep hygiene and fatigue have been associated with diminished attention and cognitive performance [18–20].

A key limitation of this study is the reliance on self-reported data from learners, which may introduce social desirability bias, as residents might have felt obligated to post more frequently given the perceived power distance in this setting. Additionally, the single-site setting restricts the generalizability of the findings.

In this study, we focused on evaluating the feasibility of this trial and did not analyze the content or quality of all 599 posts in detail. However, assessing what the residents learned during their DGIM rotation is an important aspect, and we plan to address this in our future research. Additionally, although residents were instructed that each post could be as brief as a single line, the fact that all posts spanned multiple lines suggests that their psychological safety might have been compromised when submitting reflections. Providing examples of one-line reflections in the future may help alleviate this psychological pressure.

Starting in 2025, Japan will implement working hour restrictions for physicians, including residents [21]. As a result, teaching hospitals may adopt asynchronous learning to ensure that residents receive adequate quality and quantity of educational content despite these restrictions. It is essential to mitigate whether this approach imposes hidden burdens on the learners.
